# Single Nucleotide Polymorphism (SNP)-Based Loss of Heterozygosity (LOH) Testing by Real Time PCR in Patients Suspect of Myeloproliferative Disease

**DOI:** 10.1371/journal.pone.0038362

**Published:** 2012-07-02

**Authors:** Cornelis J. J. Huijsmans, Jeroen Poodt, Jan Damen, Johannes C. van der Linden, Paul H. M. Savelkoul, Johannes F. M. Pruijt, Mirrian Hilbink, Mirjam H. A. Hermans

**Affiliations:** 1 Laboratory of Molecular Diagnostics, Jeroen Bosch Hospital, ‘s-Hertogenbosch, The Netherlands; 2 Laboratory of Pathology, Jeroen Bosch Hospital, ‘s-Hertogenbosch, The Netherlands; 3 Medical Microbiology and Infection Control, Vrije Universiteit (VU) University Medical Center, Amsterdam, The Netherlands; 4 Department of Internal Medicine, Jeroen Bosch Hospital, ‘s-Hertogenbosch, The Netherlands; 5 Jeroen Bosch Academy, Jeroen Bosch Hospital, ‘s-Hertogenbosch, The Netherlands; Sudbury Regional Hospital, Canada

## Abstract

During tumor development, loss of heterozygosity (LOH) often occurs. When LOH is preceded by an oncogene activating mutation, the mutant allele may be further potentiated if the wild-type allele is lost or inactivated. In myeloproliferative neoplasms (MPN) somatic acquisition of *JAK2V617F* may be followed by LOH resulting in loss of the wild type allele. The occurrence of LOH in MPN and other proliferative diseases may lead to a further potentiating the mutant allele and thereby increasing morbidity. A real time PCR based SNP profiling assay was developed and validated for LOH detection of the *JAK2* region (*JAK2*LOH). Blood of a cohort of 12 *JAK2V617F*-positive patients (n = 6 25–50% and n = 6>50% *JAK2V617F*) and a cohort of 81 patients suspected of MPN was stored with EDTA and subsequently used for validation. To generate germ-line profiles, non-neoplastic formalin-fixed paraffin-embedded tissue from each patient was analyzed. Results of the SNP assay were compared to those of an established Short Tandem Repeat (STR) assay. Both assays revealed *JAK2*LOH in 1/6 patients with 25–50% *JAK2V617F.* In patients with >50% *JAK2V617F*, *JAK2*LOH was detected in 6/6 by the SNP assay and 5/6 patients by the STR assay. Of the 81 patients suspected of MPN, 18 patients carried *JAK2V617F*. Both the SNP and STR assay demonstrated the occurrence of *JAK2*LOH in 5 of them. In the 63 *JAK2V617F*-negative patients, no *JAK2*LOH was observed by SNP and STR analyses. The presented SNP assay reliably detects *JAK2*LOH and is a fast and easy to perform alternative for STR analyses. We therefore anticipate the SNP approach as a proof of principle for the development of LOH SNP-assays for other clinically relevant LOH loci.

## Introduction

Neoplastic disorders may have a variety of underlying molecular and cytogenetic defects. In 2005, the involvement of the *JAK2V617F* somatic point mutation in myeloproliferative neoplasms (MPN) became apparent. *JAK2V617F* is caused by a G-to-T transversion in the *Janus Kinase 2* (*JAK2*) gene resulting in a valine-to-phenylalanine (V-F) amino acid substitution at codon 617 [1], [2], [3], [4]. JAK2 is a non-receptor tyrosine kinase involved in the JAK-STAT signalling pathway [5]. The *JAK2V617F* mutation apparently alters the activity of the autoinhibitory pseudokinase JH2 domain resulting in a constitutive activation of the JAK-STAT signalling pathway, leading to a growth factor independent cell proliferation [6]. The diagnostic value of *JAK2* mutational analysis in MPN is now well established and has been endorsed in the classification of haematological malignancies by the World Health Organisation [7].

In addition to neoplasm-promoting mutations, chromosomal imbalance may lead to loss of heterozygosity (LOH) resulting in the deletion of tumor suppressor genes. Besides this, LOH may lead to acquired partial uniparental disomy, excluding the wild-type allele of a cancer-critical gene by which the effect of the mutant oncogenic allele is further potentiated. The latter is commonly found in combination with the *JAK2V617F* mutation due to LOH of (part of) chromosome 9p [8]. A proposed mechanism which is suggested to give rise to (partial) LOH of chromosome 9p, and thereby *JAK2V617F* homozygosity, is the mitotic recombination between homologous regions of chromosome 9p in a heterozygous cell [3]. It is further speculated that the daughter cells homozygous for the *JAK2V617F* mutation gain additional proliferative advantage and outcompete the *JAK2V617F* heterozygous cells and homozygous wild-type *JAK2* cells, thereby leading to an expansion of the *JAK2V617F* homozygous subclone [3].

Polycythemia Vera (PV) patients with *JAK2V617F* often carry a homozygous subclone in contrast to Essential Thrombocythemia (ET) in which homozygosity is rare. These homozygous subclones may predominate over time. It has been proposed that it may be more common for PV patients to have a mutant allele burden of >50% than for ET patients [9]. This was confirmed in a large multicentre study during which the *JAK2V617F* mutational status in patients with PV (n = 323) and ET (n = 639) was analysed. Of the PV patients, 67.8% was found to be *JAK2V617F* heterozygous and 32.2% *JAK2V617F* homozygous. Only 2.2% of ET patients were found to be *JAK2V617F* homozygous, whereas 40.2% was *JAK2V617F*-negative and 57.6% *JAK2V617F* heterozygous [10]. Although the clinical relevance of *JAK2V617F* allele burden is not yet fully understood; a correlation between disease phenotype and the proportion of mutant alleles has been postulated [11]. Splenomegaly, vascular events and pruritus in PV and splenomegaly, arterial thrombosis and microvessel disease in ET have been clinically correlated to mutant allele burdens [12], [13]. In addition to the percentage of mutant allele burden by itself, *JAK2V617F* homozygosity (through *JAK2*LOH) is thought to give rise to a more symptomatic myeloproliferative disease (MPD) resulting in several clinical manifestations such as lower platelet counts (PV), more frequent evolution into secondary myelofibrosis, higher risks of cardiovascular events, splenomegaly and higher leukocyte counts [10], [14], [15], [16], [17]. Besides this, *JAK2V617F* homozygosity more often necessitates the use of cytoreductive therapies [10], [14]. With regard to ET, the large multicenter study showed also that ET patients harboring homozygous *JAK2V617F* mutation had a 3.9-fold higher risk of major cardiovascular events as compared to both their heterozygous and wild type counterparts [10].

Quantitative real time PCR is an excellent tool for the quantification of *JAK2V617F* mutant allele burden and is therefore of clinical importance [18]. Also, determination of the LOH status of (part of) the *JAK2* region (*JAK2*LOH) of chromosome 9p may be clinically relevant due to the possibility of pinpointing those patients carrying a *JAK2V617F* homozygous subclone, which is thought to give rise to a more symptomatic MPN. Several techniques to determine LOH status have been described, such as typing of STRs/microsatellite DNA analysis [19], [20], SNP-based pyrosequencing [19] and Affymetrix SNP microarray [8]. Although these methods provide valuable information on the LOH status, they are often not accessible for routine molecular laboratories –due to the necessity of expensive (pyro)sequencing or array equipment- and often need post-PCR processing increasing contamination risk [8], [19], [20]. Therefore we developed a *JAK2*LOH test for routine use based on real time amplification of SNPs located in and adjacent to *JAK2*.

## Materials and Methods

### Ethics Statement

The presented study was performed retrospectively using archival patient samples which were rendered anonymous before use. The Board of Directors of the Jeroen Bosch Hospital and the JBZ Scientific Advisory Board approved this study. Additionally, the JBZ Scientific Advisory Board waived the need for informed consent from the participants due to the anonymous and retrospective nature of the study. No informed consent from the involved patients was therefore obtained.

### JAK2V617F-positive Patient Cohort

To test our SNP assay for detection of *JAK2*LOH, 12 patients were selected based on *JAK2V617F*-positive mutational status. This status was determined with a quantitative *JAK2V617F* real time PCR based method using a PNA oligonucleotide for blocking the wild-type allele [18]. Patients 1 to 6 carried a *JAK2V617F* mutant allele burden of 25–50%, had a mean age of 73±8 years (mean ±1 standard deviation) and a male:female ratio of 2∶1. Patients 7 to 12 with a mutant allele burden of >50%, rendering *JAK*2LOH very likely, had an age of 77±5 years (mean ±1 SD) and a male:female ratio of 1∶1. EDTA blood samples used for *JAK2V617F* mutational status were used for LOH analysis. A non-neoplasmic archival formalin-fixed paraffin-embedded tissue (FFPE-tissue) from each patient was included to establish the germ-line profile.

Although not fully correct due to the heterogenous nature of blood tissue, results were stated as loss of heterozygosity or retention of heterozygosity (ROH) throughout this manuscript.

### Patients Suspect of MPN Cohort

Eighty-one patients, suspected for myeloproliferative disease, were included in this study. Inclusion was based on EDTA blood samples collected between December 2007 – December 2009 for *JAK2V617F* analyses in combination with the availability of archived non-neoplasm related FFPE-tissue. Mean age was 60±14 years (mean ±1 SD) with a 1∶1.3 male to female ratio. In addition to these EDTA-blood samples used for LOH analysis, the same cohort of patients was used to analyze non-neoplasm related FFPE-samples from a wide variety of tissues. These samples were originally taken for routine histological diagnostics and used in this study to determine germ-line SNP and STR profiles.

### Tissue Processing

Tissues for histological evaluation were fixed according to a routine procedure in 0.01 mol/L buffered (0.005 mol/L disodium hydrogen phosphate anhydrous and 0.005 mol/L sodium dihydrogen phosphate dihydrate, pH 7.0) 10% formalin, and processed for paraffin embedding using a Tissue-Tek VIP 5 (Sakura, Torrance, USA). The dehydration program consisted of 14 steps of 1 hour under continuous agitation, pressure, vacuum, and heating. At 40°C, two 10% formalin treatments were followed by one 70% (v/v) ethanol treatment, two 96% ethanol treatments, three 100% ethanol treatments, and two 100% xylene treatments. Paraffin embedding was done at 60°C in four 100% paraffin treatments.

### Genomic DNA Extraction from FFPE-tissues

Paraffin-embedded tissues were trimmed of paraffin excess and cut into 3 µm-thick sections. Approximately 1 to 1.5 cm^2^ of sectioned tissue (a single section or short ribbons depending on the surface per section) was put in 250 µL of digestion solution (digestion solution with proteinase K was prepared by adding 100 µL of proteinase K solution (20 mg/mL; Roche Diagnostics GmbH, Mannheim, Germany) and 10 µL of Tween 20 (Merck BV, Amsterdam, The Netherlands) to 2 mL of TE buffer (1 mmol/L EDTA, and 10 mmol/L Tris-HCl buffer, pH 8.0)) and incubated overnight at 45°C. Proteinase K was inactivated the next day by incubation at 100°C for 15–30 minutes. Afterwards, samples were centrifuged for 2 minutes at 14,000 rcf/G.

Genomic DNA was extracted from FFPE-tissues using the EasyMAG NucliSens extraction system (BioMérieux Benelux BV, Zaltbommel, The Netherlands). Two hundred µL digested sample, located beneath a paraffin cap, was added to 2 mL NucliSens lysis buffer, homogenized and incubated for 10 min. at room temperature. The mixture was then added to the EasyMAG vessel and 100 µL of diluted magnetic silica (50 µL silica +50 µL ultrapure water) was subsequently added. The DNA was extracted on the EasyMAG machine (BioMérieux Benelux BV, Zaltbommel, The Netherlands) using the “Generic 2.0.1” program. Elution was performed in 200 µL NucliSens Extraction buffer 3.

### Genomic DNA Extraction from EDTA-blood

Genomic DNA was extracted from blood using the DNA blood mini kit (Qiagen, Hilden, Germany). Two hundred µL EDTA-blood was added to 200 µL AL buffer, homogenized and incubated for 10 min. at room temperature. Two hundred µL of 96% ethanol (Merck KGaA, Darmstadt, Germany) was added. The mixture was transferred to a QIAamp column and centrifuged for 1 min. at 8,000 rcf/G. The column was put in a new collection tube, 500 µL AW1 buffer was added and centrifuged for 1 min. at 8,000 rcf/G. This procedure was repeated with 500 µL AW2 buffer and the column was centrifuged for 1 min. at 14,000 rcf/G. To remove all ethanol from the column it was put in a new collection tube and then subjected to a dry spin for 1 min. at 14,000 rcf/G. Elution was performed by adding 200 µL EL buffer, incubating for 5 min. at room temperature followed by centrifugation for 1 min. at 8,000 rcf/G.

### LOH Analysis by STR Detection

**Figure 1:**
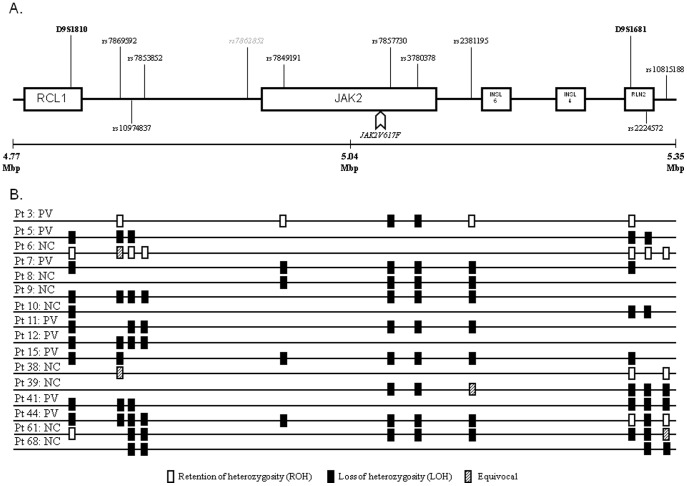
Distribution of Short Tandem Repeat (STR) and Single Nucleotide Polymorphism (SNP) markers on the *JAK2* region, along part of chromosome 9p. **A.**SNP (rs#) and STR (D9S#) marker loci within a 0.5 Mbp spanning genomic region harboring the *JAK2* gene (*JAK2* region). *RCL1* =  RNA terminal phosphate cyclase-like 1 gene, *JAK2* =  Janus Kinase 2 gene, *INSL4/6* =  insulin-like 4 and 6 genes and *RLN2* =  relaxin 2 gene. The arrow indicates the approximate position of *JAK2V617F.* **B.** STR and SNP results of *JAK2* region of 16 patients (pt). PV =  polycythemia vera, NC  =  non-classified myeloproliferative neoplasm. The boxes represent all heterozygous markers. Black boxes  =  LOH observed for this marker, white boxes  =  retention of heterozygosity observed for this marker and striped boxes  =  equivocal for this marker, homozygous markers are not shown.

Microsatellite markers D9S1810 and D9S1681 spanned a 0.5 Mbp region of chromosome 9p, including the *JAK2* gene (figure 1A) [1]. This 0.5 Mbp region –Mbp 4.8 to 5.3- is located within the minimal LOH region and downstream of the mitotic recombination breakpoint as described by Kralovics and coworkers [3].

The regions containing D9S1810 and D9S1681 were amplified. Fifteen µL of mix contained 7.5 µL 2× Amplitaq Gold FAST (Applied Biosystems, Foster City CA, USA), 0.4 µM primers (D9S1810fw: 6FAM-TAT CAA GCA AAA CTT TTT ATT GTG ATC ACA, D9S1810rv: GTT TCT CTT CTC TGA CAG CAG AGC ATC C [210 bp] D9S1681fw: VIC-AGG CAG TTG CAC AGA TAG TTA TAT ACT and D9S1681rv: GTT TCT CAG ATT CAG CCA TGT TCC AG CAT [261 bp] (ABI); The underlined sequences indicate the pig tail that was used to promote adenylated products [3]) and 50 ng of isolated DNA.

DNA isolated from both EDTA-blood (tumor) and FFPE-tissue (germline) from each patient were amplified in the same run using a Veriti FAST Thermal Cycler (Applied Biosystems, Foster City CA, USA) for 12 min. at 94°C followed by 10 cycles of 15 sec. at 94°C, 15 sec. at 60°C, 30 sec. at 72°C, followed by 25 cycles of 15 sec. at 89°C, 15 sec. at 60°C, 30 sec. at 72°C and a final elongation step of 10 min. at 72°C.

Prior to capillary electrophoresis, PCR products were diluted in ultrapure water to obtain a fluorescence signal between 400 and 8000 Fluorescent Units. Capillary electrophoresis and the Fluorescence Unit measurement of each PCR product was performed on a 3130 Genetic Analyzer (Applied Biosystems, Foster City CA, USA) in Hi-Di formamide together with a LIZ-600 size marker (Applied Biosystems, Foster City CA, USA). Allele ratios of tumor material were compared to allele ratios of germline DNA, using Genemapper Software Version 4.0 (Applied Biosystems, Foster City CA, USA).

### LOH Analysis by SNP Detection

Ten predesigned TaqMan® SNP Genotyping Assays (Applied Biosystems, Foster City CA, USA) were used according to the manufacturer’s instructions. Twenty-five µL of PCR, using the in house made JBZ 4× master mix contained, 20 mmol/L Tris-HCl, pH 8.4, 50 mmol/L KCl, 3 mmol/L MgCl_2_ (prepared from 10× PCR buffer and 50 mmol/L MgCl_2_ solution delivered with Platinum Taq polymerase), 0.75 U of Platinum Taq polymerase (Invitrogen BV, Breda, The Netherlands), 4% glycerol (molecular biology grade; Calbiochem, VWR International BV, Amsterdam, The Netherlands), 200 µmol/L of each dNTP (Invitrogen BV), 0.5 µL of Rox reference dye (Invitrogen BV), 1.25 µL of pre-developed assay reagent from the Assays-on-Demand SNP genotyping products (Applied Biosystems, Foster City CA, USA) containing two primers and two MGB TaqMan probes (5′ VIC for allele 1, 5′ FAM for allele 2 and a 3′ black hole quencher for both alleles), and 11.25 µL of target DNA. Real time PCR was performed in three different ABI Prism 7500 FAST SDS (ABI) machines for 1 minute at 95°C, followed by 45 cycles of 3 seconds at 95°C and 30 seconds at 60°.

### Statistical Analysis SNP LOH Detection

To determine and visualize the distribution of delta Rn VIC/FAM ratios (ratio allele 1/allele 2), box-and-whisker plots were generated for each SNP based of the EDTA-blood samples from the heterozygous patients –based on the germline profile generated using the corresponding FFPE-tissues– from the patient cohort that were negative for the *JAK2V617F* mutation (63/81). The box extends from the 25^th^ to 75^th^ percentile of observations, representing the interquartile range (IQR). The bars/whiskers define the +1.5 IQR and −1.5 IQR and indicate the cut off value for normal ratio distribution. Therefore, ratios plotted within 1.5–3× IQR are outliers and were considered equivocal with regard to *JAK2*LOH status. Ratios plotted ≥3× IQR are extremes and were considered to be representative for *JAK2*LOH. All ratios with an IQR of <1.5 follow normal distribution and were interpreted as *JAK*2 ROH.

### Leukocyte, Thrombocyte and Erythrocyte Counts

Leukocyte, thrombocyte and erythrocyte counts were routinely determined directly after sampling by the SE9500/XE2100 (Goffin Meyvis, Etten-Leur, The Netherlands) analyzers.

The cell counts were firstly judged for fit to the normal distribution by using stem-and-leaf plots and quantile-quantile plots. As our data did not follow a normal distribution, Mann-Whitney U tests were performed for comparison of the cell counts between the *JAK2*ROH and *JAK2*LOH group.

## Results

### STR and SNP Selection

STRs D9S1810 and D9S1681 span the 0.5 Mbp minimal 9pLOH region, incorporating the *JAK2* gene (figure 1A), and have been used before by Kralovics and coworkers to investigate 9pLOH [3].

Ten SNPs were selected *in silico* using SNP browser software Version 4.0 (ABI). Selection was based on allele frequencies of around 0.5 and location within or near the *JAK2* gene covering the 0.5 Mbp 9p region in between D9S1810 and D9S1681 (figure 1A and table 1). For real time PCR based amplification and SNP detection, TaqMan® SNP Genotyping Assays from Applied Biosystems were used. During technical validation, one (SNP rs7862852) of the ten SNP assays showed cross reactivity of the FAM MGB probe with allele 1 (VIC) and poor amplification of allele 2 (FAM) in the presence of allele 1. Therefore, allele calling could not be performed and TaqMan® SNP Genotyping Assay for rs7862852 was excluded from LOH analyses. The other 9 SNP assays generated reliable allele calls and were therefore suitable for inclusion in the *JAK2*LOH panel (figure 1A and table 1).

**Table 1:** Panel of selected Single Nucleotide Polymorphisms (SNPs).

SNP-ID(hCV)	SNP-ID(rs)	Base(9p)	SNP	Minor allele frequency			
				CEU	CHB	JPT	YRI
29340651	7869592	4,866,301	T/C	0.46	0.40	0.35	0.47
2008226	10974837	4,885,391	A/G	0.48	0.44	0.49	0.46
657006	7853852	4,879,126	C/G	0.46	0.49	0.47	0.49
*34291956* *	*7862852*	*4,962,398*	*C/T*	*0.45*	*0.46*	*0.35*	*0.33*
2008287	7849191	4,978,761	C/T	0.50	0.48	0.41	0.19
29340600	7857730	5,074,049	T/G	0.47	0.42	0.45	0.11
27480690	3780378	5,102,288	T/C	0.47	0.46	0.50	0.45
16225021	2381195	5,135,887	T/C	0.49	NA	NA	NA
28001006	2224572	5,278,707	A/T	0.37	0.43	0.49	0.44
31941380	10815188	5,344,681	T/C	0.40	0.41	0.48	0.30

SNP-ID (hCV)  =  Celera SNP ID. SNP-ID (rs)  =  reference SNP ID number. Base (9p)  =  the nucleotide position on chromosome 9(p). The particular nucleotide variation is referenced in “SNP”-column. Minor allele frequencies are indicated for different populations: CEU, CEPH (Centre d’Etude du Polymorphisme Humain) from Utah; CHB, Chinese from Beijing; JPT, Japanse from Tokyo and YRI, Yoruba from Ibadan Nigeria.

* SNP rs7862852 is excluded from the panel of recommended SNPs. NA  =  not applicable.

### Patient Cohorts

Two patient cohorts were used in this study: a cohort of 12 *JAK2V617F*-positive patients and a cohort of 81 patients suspect of MPN.

**Figure 2:**
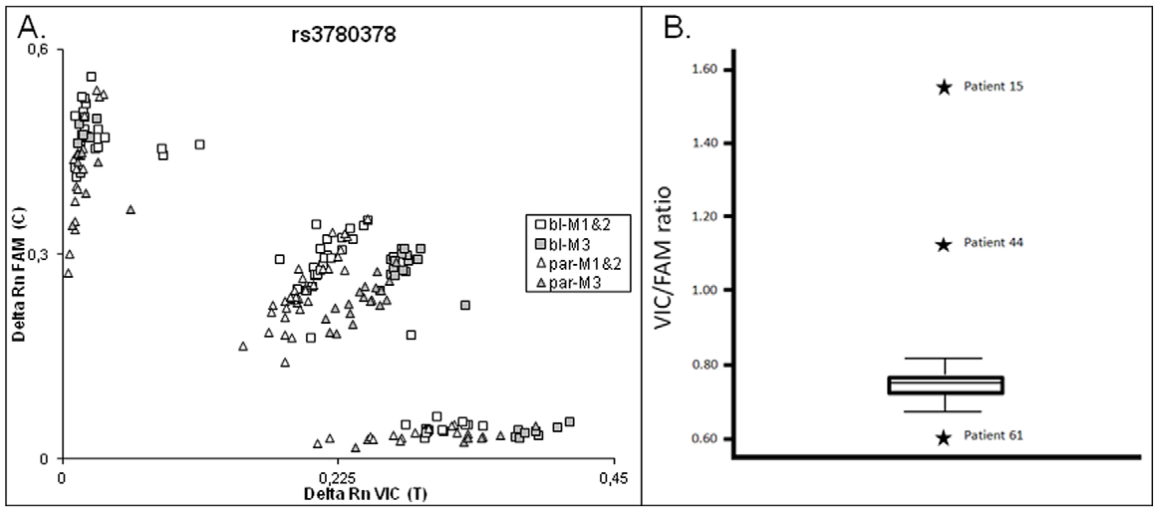
Visualization of FAM/VIC ratios generated by representative SNP rs3780378 **A.**Scatterplot of SNP rs3780378 analyses of paired EDTA-blood and formalin-fixed paraffin-embedded (FFPE)-tissues of 93 patients (12 patients from *JAK2V617F*-positive cohort and 81 sample pairs from the MPN suspect patient cohort). X-axis: Delta Rn VIC (T  =  Thymine), Y-axis: Delta Rn FAM (C  =  Cytosine). White squares (bl-M1&2)  =  EDTA-blood samples amplified using ABI7500FAST machines 1 and 2, grey squares (bl-M3)  =  EDTA-blood samples amplified using ABI7500FAST machine 3, white triangles (par-M1&2)  =  FFPE-tissue samples amplified using ABI7500FAST machines 1 and 2 and grey triangles (par-M3)  =  FFPE-tissue samples amplified using ABI7500FAST machine 3. **B.** Representative box-and-whisker plot generated using the SNP rs3780378 VIC/FAM ratios of the EDTA-blood samples of all heterozygous patients from the patient cohort analyzed with ABI7500FAST machines 1 and 2. Stars represent extremes (>3× IQR  =  *JAK2*LOH).

FAM/VIC ratios were plotted per SNP in a scatterplot. Figure 2A shows the scatterplot of SNP rs3780378 for paired paraffin and blood samples of all patients in both cohorts. The data obtained for SNP rs3780378 are representative for the entire SNP panel. Four clusters –homozygous VIC, homozygous FAM, heterozygous FAM/VIC ABI7500FAST machine 1 and 2 and heterozygous FAM/VIC ABI7500FAST machine 3- were observed. FAM/VIC ratios plotted outside these clusters were considered to represent LOH or to be equivocal as was statistically determined using box-and-whisker plots (figure 2B). In addition, a FAM/VIC ratio of a blood sample from a heterozygous patient present in a homozygous cluster was considered to represent LOH.

### Statistical Analysis SNP LOH Detection

Box-and-whisker plots were generated, based on the VIC/FAM ratios from EDTA-blood samples of all heterozygous patients from the patient cohort suspect of MPN that were negative for the *JAK2V617F* mutation (63/81), for all SNP assays (data not shown). Data from the *JAK2V617F*-positive cohort samples that were selected based on their mutant allele burdens of 25–50% and >50% *JAK2V617F* and therefore more likely to contain *JAK2*LOH were not included. Inclusion of *JAK2V617F*-positives would lead to a larger variety in VIC/FAM ratios and therefore a biased IQR. This would influence outlier (equivocal) and extreme (*JAK2*LOH) calling. VIC/FAM ratios generated with the *JAK2V617F*-positive patients were called (*JAK2*ROH, equivocal or *JAK2*LOH) using the measured IQRs of the *JAK2V617F-*negative patients from the patient cohort.

One representative box-and-whisker plot generated using SNP rs3780378 is shown in figure 2B. Based on these calculations, patient 15, 39, 44 and 61 were found to be extremes (*JAK2*LOH) as additionally presented in figure 1B.

**Table 2:** Cross table of *JAK2* region loss of heterozygosity results of the *JAK2V617F*-positive patient cohort, generated by the Short Tandem Repeat (STR) assay and the Single Nucleotide Polymorphism (SNP) based assay.

	SNP *JAK2 *LOH assay		
STR *JAK2 *LOH assay	*JAK2*LOH	*JAK2*ROH	Total
*JAK2*LOH	6^A^	0	6
*JAK2*ROH	1^B^	3^C^	4
Total	7	3	10

A =  patient (pt) 5, 7, 9, 10, 11 and 12,

B =  pt 3*, ^C^ =  pt 1, 2 and 6**.

* LOH observed for a small region of the *JAK2* gene, in proximity of the *JAK2V617F* codon.

** rs7869592 equivocal, other 4 SNPs indicative of ROH.

*JAK2*LOH  =  loss of heterozygosity on the *JAK2* region, *JAK2*ROH  =  retention of heterozygosity on the *JAK2* region.

### JAK2V617F-positive Patient Cohort

The cohort of 12 *JAK2V617F*-positive patients was composed with samples containing mutant allele burdens of 25–50% (patients 1–6) and burdens above 50% (patients 7–12). The results of STR vs. SNP LOH analyses are summarized in cross table 2. Patients 1, 2 and 4 showed STR and SNP results consistent with ROH of the *JAK2* region. Patients 5, 7, 9, 10, 11 and 12 showed *JAK2*LOH in both assays. In patient 3 discrepant results were found in STR vs. SNP testing (ROH/LOH). The STR assay results were indicative of *JAK2*ROH, whilst the SNP profile revealed different results among the various SNPs. SNPs rs7857730 and rs3780378 revealed the presence of one of the two germ line alleles, while the 3 other informative SNPs were found to be heterozygous. Overall results for patient 5 were ROH/ROH. However, SNP rs7869592 result was found to be equivocal, while the 4 other SNPs showed ROH and the remaining 4 SNPs were non-informative (table 2). LOH of the *JAK2* region was additionally shown in patient 8 using the SNP assay while STR results were non-informative (figure 1B and table 3). In patient 4, SNP rs7869592 was heterozygous but was found to be equivocal, STR results were indicative for ROH.

**Table 3:** Cross table of equivocal and non-informative results, generated by the Short Tandem Repeat (STR) assay and the Single Nucleotide Polymorphism (SNP) based assay.

	SNP *JAK2*LOH assay				
STR *JAK2*LOHassay	*JAK2*LOH	*JAK2*ROH	Non-informative	Equivocal	Total
*JAK2*LOH	NA	NA	0	0	0
*JAK2*ROH	NA	NA	3^E^	2^F^	5
Non-informative	2^G^	7^H^	0	0	9
Equivocal	0	0	0	0	0
Total	2	7	3	2	14

E =  pt 32, 55 and 93,

F =  pt 4 and 38,

G =  pt 8 and 68,

H =  pt 13, 20, 22, 24, 25, 59 and 69.

*JAK2*LOH  =  loss of heterozygosity on *the JAK2* region, *JAK2*ROH  =  retention of heterozygosity on the *JAK2* region. Results were indicated as non-informative due to homozygous STR/SNP profiles in the formalin-fixed paraffin-embedded tissues. NA  =  not applicable.

### Patients Suspect of MPN Cohort

Eighteen patients harbored the *JAK2V617F* mutation, whereas 63 patients did not. The *JAK2*LOH/ROH results for both groups, as generated by SNP and STR analysis, are summarized in cross table 4. Equivocal and non-informative results are summarized in table 3.

**Table 4:** Cross table of *JAK2* region loss of heterozygosity results of the patients suspect of MPN cohort, generated by the Short Tandem Repeat (STR) assay and the Single Nucleotide Polymorphism (SNP) based assay.

	SNP *JAK2*LOH assay		
STR *JAK2*LOH assay	*JAK2*LOH	*JAK2*ROH	Total
*JAK2*LOH	5^D^	0	5
*JAK2*ROH	0	64	64
Total	5	64	69

D =  patients 15, 39, 41, 44 and 61.

*JAK2*LOH  =  loss of heterozygosity on the *JAK2* region, *JAK2*ROH  =  retention of heterozygosity on the *JAK2* region. MPN  =  myeloproliferative neoplasm.

Within this cohort, 64 patients were found to generate ROH results in both assays (ROH/ROH). Patients 15, 39, 41, 44 and 61–all *JAK2V617F*-positive- yielded results indicative of JAK2LOH in both assays (figure 1B). Patient 68 displayed *JAK2*LOH in the SNP assay, whilst the STR assay was non-informative due to homozygosity for both markers. Patient 38 yielded equivocal results using SNP rs7869592, while SNP rs10815188 and STR D9S1810 results were indicative of ROH. Patients 13, 20, 22, 24, 25, 59 and 69 were found to be non-informative with the STR assay, while SNP analysis showed *JAK2*ROH. SNP profiles of patients 32, 55 and 93 were considered non-informative due to an incomplete SNP profile (4, 7 and 5 out of 10 SNPs, respectively) of the reference FFPE-sample (due to low DNA loads) in combination with being homozygous for all interpretable SNPs. These patients showed *JAK2*ROH in the STR assay (table 3).

### Leukocyte, Thrombocyte and Erythrocyte Counts

Mean cell counts (±1 SD), due to the retrospective nature of this study available for 24 *JAK2V617F*-positive patients, for the *JAK2*LOH (n = 12 [including patient 3]) vs. the *JAK2*ROH (n = 12) group were: 18.7±14.9×10^9^ vs. 10.6±4.1×10^9^ leukocytes/L, 460±266×10^9^ vs. 688±390×10^9^ thrombocytes/L and 6.00±1.38×10^12^ vs. 4.58±1.05×10^12^ erythrocytes/L.

Leukocyte and erythrocyte counts were significantly higher (p-value  = 0.045 and 0.007) in the *JAK2*LOH group than in the *JAK2*ROH group. Thrombocyte counts did not differ significantly between the two groups (p-value  = 0.068).

## Discussion

We have developed a SNP based assay, which is easy to perform and reliably detects *JAK2*LOH. Ten SNPs located on 9p within the *JAK2* region were selected *in silico* based on incidence around 0.5 in Caucasian (CEU, CEPH (Centre d’Etude du Polymorphisme Humain) from Utah), Chinese (CHB, Chinese from Beijing), Japanese (JPT, Japanese from Tokyo) and African (YRI, Yoruba from Ibadan Nigeria) populations. Technical validation of the accompanying SNP assays revealed that nine SNP assays fulfilled the criteria for inclusion -clear distinction of homo−/heterozygous clusters, allele frequency in investigated population around 0.5 (data not shown) and sufficient sensitivity- in the *JAK2*LOH/ROH panel. SNP rs7862852 was excluded from the recommended panel of SNP markers due to poor performance.

A second technical issue with regard to the SNP assay was encountered when three different ABI7500FAST systems were employed. Two distinct groups of homozygotes were observed for machine 1&2 vs. machine 3 (figure 2). For each ABI7500FAST system all dyes employed (including FAM and VIC) are calibrated individually once every three months in our diagnostic setting. Variations introduced during such a calibration are inevitable as well as those caused by differences in light intensity generated by the integrated lamp which is replaced after 2,000 burning hours. These technical issues may induce inter-machine variability. To prevent misinterpretation of *JAK2*LOH/ROH status, we recommend analyzing EDTA-blood samples and corresponding FFPE-tissue on one ABI7500FAST as a control within the same run.

When interpreting the results with regard to *JAK2*LOH and ROH status, it should be taken into account that blood is a heterogeneous tissue. The demonstrated technique will therefore only detect *JAK2*LOH in those patients where the homozygous subclone is abundant enough. The assess the range in which homozygous subclones are abundant enough to be detected by the SNP assay, a serial dilution of DNA of a healthy homozygous patient and DNA of a healthy heterozygous patient (for SNP rs7849191) was made and tested. The 5%, 10% and ≥20% (20, 30, 40 and 50%) dilutions yielded results that were interpreted using the generated cut off values as “ROH”, equivocal with regard to “LOH status” and indicative of “LOH”, respectively (results not shown). Indicating a sensitivity of at least 20% homozygous cells in a heterozygous background using representative SNP rs7849191.

With regard to the interpretation of the STR results, difficulties were encountered when analyzing the data automatically. When allelic ratios were called by the Genemapper Software Version 4.0 (ABI), LOH/ROH calls were found to be doubtful in cases where DNA loads were aberrant -low loads observed in several FFPE-tissues and high loads in some blood samples-. The software did not take into account that signals >8,000 or with very low fluorescent units negatively influenced allelic ratios, resulting in false LOH calls. Therefore, an additional manual analysis of all STR results was performed to determine STR *JAK2* status. To assure that manual interpretation was objective, an LOH index was calculated. This index was calculated by determining the intensity of the PCR products, which was used to calculate the intensity ratio between the peaks of both alleles in the tumor sample. Dividing this ratio by the same ratio found in the normal tissue yielded the LOH index. When this LOH index was below 0.75, this was interpreted as LOH, whereas an LOH index of >0.75 was interpreted as ROH.

Considering the STR assay to be the golden standard, the SNP assay’s sensitivity and negative predictive value were 100% for both the *JAK2V617F*-positive patient cohort and the patients suspect of MPN cohort. Positive predictive values of the SNP assay were 85.7% and 100% and the specificity was 80% and 100% in the respective cohorts. Patient 3 showed no signs of *JAK2*LOH using the STR assay. Analyses of SNPs close to both STR loci also showed ROH. However, two SNPs in between the two STR loci in relative close proximity to *JAK2V617F* (rs7857730 and rs3780378 10–15 Kb and 35–50 Kb downstream, resp.) were not indicative of ROH. Rs7857730 and rs3780378 clearly showed *JAK2*LOH. It is likely that a small deletion, point mutation or a small recombination event around rs7857730 and rs3780378 accounts for these observations.

Of the 81 patients in the patient cohort suspect of MPN, *JAK2*LOH was found in 6 patients (7%), i.e. one-third (6/18) of the *JAK2V617F*-positive patients. Amongst the *JAK2V617F*-negative patients, no cases of *JAK2*LOH were found.

That *JAK2*LOH was found in 2/5 patients from the *JAK2V617F*-positive cohort with a *JAK2V617F* mutant allele burden below 50% is in line with the findings of Scott and coworkers, describing that a large number of PV patients harbor homozygous subclones, which may predominate over time [9]. Moreover, it indicates that next to JAK2V617F quantification the determination of *JAK2*LOH status provides additional information and could be of value.

In patient 4, rs7869592 was the only informative SNP and was found to be equivocal. Patient 38–positive for *JAK2V617F*- yielded an equivocal result for SNP rs7869592, SNP rs10815188 and D9S1681 were representative of *JAK2*ROH. All other markers were non-informative due to homozygosity. The overall result was therefore concluded to be equivocal for patients 4 and 38 (figure 1B, table 3). Patient 44 yielded results indicative of *JAK2*LOH for D9S1810 and 8/9 SNPs, whereas D9S1681 and rs10815188 yielded results indicative of *JAK2*ROH (figure 1B). The mechanism behind this observation is unclear. A possible explanation could be an inversion of the D9S1681/rs2224572 locus that might have arisen during the process of recombination. In patient 61, the result from marker D9S1681 was indicative of *JAK2*LOH, whereas D9S1810 yielded a *JAK2*ROH result. Results from 6/9 SNPs were indicative of *JAK2*LOH, the result from one SNP was equivocal (figure 1B). We presume that this aberrant observation is due to mutation of the concerning *JAK2* region.

In both patient cohorts the STR assay resulted in a total of 9 non-informative homozygous profiles, of which patient 8 and 68 were found in the SNP assay to harbor cells with *JAK2*LOH. Analysis of the cohorts using the SNP assay resulted in 3 non-informative homozygous profiles, of which the STR results were representative of *JAK2*ROH. Thus the SNP assay has a higher success rate than the STR assay in this population. Caution is however advised when handling samples with low DNA loads. Additionally, the SNP assay provides more detailed information with regard to ROH/LOH status of the JAK2 gene and surrounding loci.

In total, in 14 patients the SNP assay results were indicative of *JAK2*LOH (including patient 3, where a small part of the *JAK2* region was affected; figure 1B and table 2) with an overall mean age of 74±7 years and a male:female ratio of 1.00∶0.75. The 30 *JAK2V617F*-positive patients consisted of 8 PV patients, 7 ET patients, 1 IMF patient and 14 patients with a non-classified MPN. The occurrence of *JAK2*LOH amongst the different disease phenotypes was: PV 100% (n = 8), ET 0% (n = 7), IMF 0% (n = 1) and non-classified MPN 42.9% (n = 14). Similar distributions were previously found showing that *JAK2*LOH is a common event in PV and the contrary, is very rare in ET and IMF [9], [10], [11], [12], [13], [14], [15], [16], [17]. The fact that within the group of *JAK2V617F*-positive patients, leukocyte and erythrocyte counts were significantly higher in patients with *JAK2*LOH in comparison to patients with *JAK2*ROH may indicate a more symptomatic disease in the first group. This is in line with previous findings where the higher mutant allele burden was found to be correlated to lower platelet counts, higher incidence of splenomegaly and larger spleen size, cytoreductive therapy needed in a greater number of patients and higher white blood cell counts in PV and ET. A higher incidence of thrombotic events is also observed in PV and ET, particularly with mutant allele burdens above 50%. Significantly higher chances of fibrotic transformation have been observed in both PV and ET [10], [14], [15], [16], [17]. Additionally, MPN in patients 10 and 44 with *JAK2*LOH evolved into leukemia; patient 10 suffered from AML and patient 44 suffered from B- CLL in combination with AML and subsequently deceased. No leukemic transformation was observed in the *JAK2*ROH group.

Because the SNP assay relies on real time PCR, the technique is fast, easy to perform and requires less hands-on time in comparison to STR analysis –1.5 hours vs. 3 hours-. In addition, the SNP assay does not require the use of expensive sequencing equipment, is therefore more easily accessible for smaller routine molecular diagnostic laboratories. No post-PCR processing is necessary, diminishing contamination risks. Besides the current application of *JAK2*LOH testing, the demonstrated SNP technique may prove useful as a diagnostic strategy for determination of LOH status in other neoplastic entities such as 18q LOH in colorectal cancer [21], [22], [23]. In addition to the in this study described use of archival FFPE-tissues to generate germline SNP profiles, real time PCR based SNP profiling was described to be suitable in combination with buccal swabs [24], providing more-easy-to-obtain reference material.

In conclusion, we have developed a novel and easily accessible SNP based assay to reliably determine *JAK2*LOH status.
